# Peptide biomarkers used for the selective breeding of a complex polygenic trait in honey bees

**DOI:** 10.1038/s41598-017-08464-2

**Published:** 2017-08-21

**Authors:** M. Marta Guarna, Shelley E. Hoover, Elizabeth Huxter, Heather Higo, Kyung-Mee Moon, Dominik Domanski, Miriam E. F. Bixby, Andony P. Melathopoulos, Abdullah Ibrahim, Michael Peirson, Suresh Desai, Derek Micholson, Rick White, Christoph H. Borchers, Robert W. Currie, Stephen F. Pernal, Leonard J. Foster

**Affiliations:** 1 Department of Biochemistry & Molecular Biology, and Centre for Sustainable Food Systems, University of British Columbia, Vancouver, BC Canada; 2 Agriculture & Agri-Food Canada, Beaverlodge Research Farm, Beaverlodge, AB Canada; 3 Alberta Agriculture and Forestry, Lethbridge, AB Canada; 4 Kettle Valley Queens, Grand Forks, BC Canada; 5 University of Victoria-Genome British Columbia Proteomics Centre, Victoria, BC Canada; 6 Department of Horticulture, Oregon State University, Corvallis, OR USA; 7 Department of Entomology, University of Manitoba, Winnipeg, MB Canada; 8 Department of Statistics, University of British Columbia, Vancouver, BC Canada; 9 Department of Biochemistry and Microbiology, University of Victoria, Victoria, British Columbia Canada; 10 Lady Davis Institute, Jewish General Hospital, McGill University, Montreal, Quebec Canada; 11 Gerald Bronfman Department of Oncology, Jewish General Hospital, Montreal, Quebec Canada

## Abstract

We present a novel way to select for highly polygenic traits. For millennia, humans have used observable phenotypes to selectively breed stronger or more productive livestock and crops. Selection on genotype, using single-nucleotide polymorphisms (SNPs) and genome profiling, is also now applied broadly in livestock breeding programs; however, selection on protein/peptide or mRNA expression markers has not yet been proven useful. Here we demonstrate the utility of protein markers to select for disease-resistant hygienic behavior in the European honey bee (*Apis mellifera* L.). Robust, mechanistically-linked protein expression markers, by integrating *cis-* and *trans-* effects from many genomic loci, may overcome limitations of genomic markers to allow for selection. After three generations of selection, the resulting marker-selected stock outperformed an unselected benchmark stock in terms of hygienic behavior, and had improved survival when challenged with a bacterial disease or a parasitic mite, similar to bees selected using a phenotype–based assessment for this trait. This is the first demonstration of the efficacy of protein markers for industrial selective breeding in any agricultural species, plant or animal.

## Introduction

European honey bees are a keystone species in agriculture as many crops depend on them for pollination and increased yield^1^. Honey bee colonies have been dying at increased rates over the past decade, largely due to increased pressure from diseases and pests, as well as pesticide use and habitat loss^2, 3^. The most important pests and pathogens of bees are currently controlled with acaricides, antibiotics and antimycotics, but emerging resistance to these treatments may be partially responsible for the higher level of colony losses seen over the past several years^4^. These exogenous treatments can also leave residues in the hive^5^ and honey. Thus, the most sustainable, long-term solution for bee health is the development of selective breeding programs that can enrich natural disease resistance mechanisms. However, selective breeding in *A. mellifera* is particularly challenging because many traits of interest are only expressed at the colony level^6^ (e.g honey production, overwintering success). In addition, their haplo-diploid sex determination system^7^, difficulties in storing germplasm^8^, the requirement for heterozygosity at the complementary sex determination locus^9^ that severely limits in-breeding, and the tendency for queens to mate with up to twenty different drones^10^ mean that continual selection is required to maintain stock.

Bees do, however, have some effective disease-resistance traits, which also happen to be highly polygenic: one example is the social immunity function known as hygienic behavior. We use the term hygienic behavior in this manuscript specifically to refer to the hygienic removal of freeze-killed brood (FKB). Bees exhibiting this behavior are more efficient at removing dead, diseased, or dying brood from the hive^11, 12^, enabling them to resist brood pathogens such as American foulbrood (AFB; caused by the bacterium *Paenibacillus larvae*) and chalkbrood (*Ascophaera apis)* that would otherwise reduce productivity or even kill the colony, and also conferring partial resistance to parasites such as the *Varroa* mite (*V. destructor*)^13^. A closely related but distinct trait known as *Varroa*-sensitive hygiene enables bees to detect and disrupt the life cycle of reproductive female *Varroa* mites^14^. Although the relationship between these traits is still under investigation, the FKB response and *Varroa*-sensitive hygiene are heritable^15^ and can therefore be selectively bred for.

QTLs and SNPs have been linked to each behavior^16, 17^ enabling their potential use in marker-assisted selective breeding. Historically, genomic markers have been favoured for marker-assisted selection (MAS) because of their stability and reliability, whereas expression markers, such as the levels of transcripts or proteins, are typically thought to be too variable and dependent on environment for use in MAS. Even a closely linked DNA feature may not be sufficient for MAS in honey bees: *A. mellifera* has one of the highest recombination rates (~32 cM/Mb) known among animals, and this will rapidly break down inter-allele linkage through repeated rounds of meiosis^18^. Conversely, causally-linked expression markers should be more robust to recombination because their presence is required for the trait.

Here we use a panel of protein markers identified through a multi-generational study^19^ to guide selective breeding of disease-resistance traits in honey bees through three generations. By the third generation, bee stocks selected through MAS were able to resist disease as effectively as bees raised through conventional selective breeding using standard field tests, with no detectable loss of other desirable traits such as honey production. This is the first successful use of expression markers for MAS that we are aware of.

## Results and Discussion

**Figure 1:**
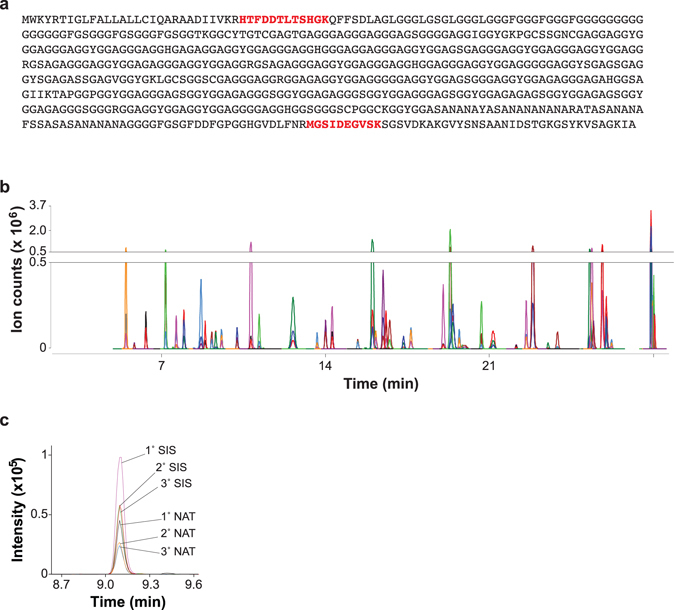
Multiple reaction monitoring assays for markers of disease resistance. (**a**) Amino acid sequence of gi:110761334, Glycine-rich cell wall structural protein-like protein, one of the markers of hygienic behavior. The two peptides identified in the initial discovery are highlighted in red; these same two peptides were targeted with multiple reaction monitoring assays here. (**b**) Overlaid chromatograms of the three selected transitions for the stable isotope-labelled forms of all fifty-five peptides listed in Table 1 for the fifteen proteins comprising the biomarker panel. (**c**) Transitions for the stable isotope standard (SIS) and natural (NAT) forms of MGSIDEGVSK from Glycine-rich cell wall structural protein-like protein. The primary (1°) transition of each peptide was used for quantitation, while the secondary and tertiary transitions were used to confirm specificity.

We have previously identified seven proteins in adult worker bees’ antennae whose expression is tightly linked to hygienic behavior^19^. To complete a comprehensive panel of markers for use in selective breeding, we added six more proteins derived from the same data, including four that showed tight correlation with *Varroa*-sensitive hygiene and grooming behavior, and two more also linked to hygienic behavior. Of the latter, one was missing in an initial dataset and therefore failed to meet the inclusion criteria we used, and the other (Fig. 1a) had been ‘lost’ due to an unrealized change in accession number between protein database versions. The four *Varroa-*related markers were identified by a correlation analysis of protein levels, using the same discovery proteomics approach we previously described^19^, and *Varroa* related field- based assays^20^. These thirteen biomarkers were complemented by two additional ‘housekeeping’ proteins, α-spectrin and β-tubulin, that showed zero correlation with any behaviors (Dataset 1) to serve as loading controls. The biomarker panel had originally been discovered through untargeted, data-dependent liquid chromatography-tandem mass spectrometry but a less stochastic detection method was required for scanning hundreds of samples. Therefore, multiple reaction monitoring (MRM) assays^21^ were developed for up to five peptides from each protein (Dataset 1, Fig. 1b), with priority given to peptides we had observed in the discovery of these biomarkers^19^. The use of stable isotope-labelled standard peptides of known concentrations allowed quantification of each peptide in protein extracts from worker bee antennae (Fig. 1c).

**Figure 2:**
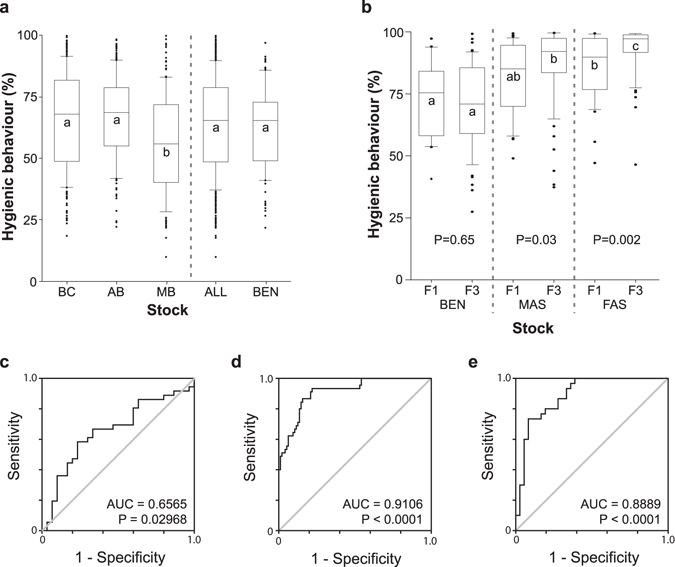
Starting distributions and enrichment of hygienic behavior. BEN = benchmark, MAS = Marker-assisted selection, FAS = Field-assisted selection. (**a**) 90/10 box-and-whisker plots with median values of the hygienic behavior scores from all colonies in initial survey across Western Canada, in British Columbia (BC), Alberta (AB), Manitoba (MB)(left section) (Means of groups with the same letter are not different from each other, Tukey P < 0.05), all colonies together (ALL), and the randomly selected starting benchmark population (BEN). ‘All’ is statistically identical to BEN (p = 0.21, Analysis of Means Test). (**b**) The distribution of hygienic behavior in the F1 and F3 generations of the benchmark population (left section, BEN, no statistical difference between F1 and F3, P = 0.65, contrast), the colonies selected by the biomarker panel (middle section, MAS, F3 > F1, p = 0.03, contrast), and the freeze-killed brood assay (right section, FAS, F3 > F1, p = 0.002, contrast). Within each generation, means of groups with the same letter are not different from each other, Tukey P = 0.05). Bottom: Receiver operating characteristics illustrating the performance of the F1 (**c**), F2 (**d**) and F3 (**e**) marker panels used for MAS.

To identify a robust initial breeding population of honey bees that was not already enriched in disease-resistance behaviors, we surveyed hygienic behavior of 635 colonies from thirty-eight commercial beekeeping operations across western Canada in 2011. For this initial survey, we gave priority to beekeeping operations that bred their own bees to ensure that the bees were well-adapted to the local climate^22^ and representative of stock being bred and used in western Canada. All beekeepers donated or sold a subset of the tested queens to incorporate into the selection program. The scores using the freeze-killed brood assay in this initial survey varied regionally and ranged from 9.8% to 100% (see Dataset 2.1 for data on individual populations). Such a large variation in the hygienic behavior response has been observed in other populations tested^23^. The median hygienic behavior response in our initial survey was 64% (Fig. 2a), matching levels of trait expression observed previously among unselected populations in a smaller survey within the same region^24^. The contribution of each of the 38 source populations to the initial stock (Dataset 2.1) ranged from 1% to a high of 6%. Using diverse source populations may have increased levels of genetic variance in the starting population beyond those normally found within one population and thereby may have contributed to a higher level of response to selection.

For selection using markers, nurse bees from 468 of the 635 colonies were dissected for quantitation of peptide markers in their antennae (Dataset 3). From the colonies surveyed, two to four colonies were randomly selected from each beekeeping operation (for a total of 100 queens) to serve as benchmark, unselected stock (Fig. 2a): these (BEN) were statistically indistinguishable from the wider surveyed population (ALL). In addition, we selected queens from an additional 100 colonies, each with the highest scores in their apiaries. Their hygienic behavior scores ranged from 38% to 100% with a mean score of 85%. We moved these queens to two breeding locations in southern British Columbia and introduced them into colonies.

Over the next two years we reared three successive generations (F1 and F2 in 2012, F3 in 2013) from this initial population using the response of parental colonies in each of two ways: (1) the freeze-killed brood assay^11^ to quantify hygienic behavior as a positive control (FAS, field-assisted selection), and (2) the levels of the best-performing subset of the peptide markers in Supplemental Table 3 (MAS, marker-assisted selection). For the selective breeding, the F1 and F2 queens were generated by instrumental insemination of virgin queens reared from the selected colonies using semen pooled from a random collection of drones from all the selected colonies in the appropriate stock. The same pooling of semen was accomplished for F3 queens by closed breeding in isolated mountain valleys in southeastern British Columbia where no known contaminating drone sources existed. In addition to these selective matings, benchmark stock (BEN) was maintained through unselected open mating, as a control.

Colonies selectively bred for hygienic behavior using FAS, employing the FKB assay showed the greatest enrichment in this behavior over three generations (Fig. 2b). Notably, selective breeding based on the panel of protein markers (MAS) was also effective for enriching hygienic behavior, demonstrating the potential of this technique in selective breeding.

By measuring hygienic behavior in the colonies bred using MAS we could also monitor the specificity and sensitivity characteristics of the biomarker panels; while there was a statistically significant improvement detectable even in F1, by F2 there was a very marked improvement that was little changed in the F3 (Fig. 2c,d,e). The distribution of hygienic behavior in the unselected BEN colonies, however, was unchanged between the starting group and the final population (Fig. 2b, left-most plot).

Evaluation of the contribution of each source population to the selected populations showed that, although there was a diversity of sources represented in the final populations, two were represented in higher proportions (Dataset 2.2). We also estimated the realized heritability for FAS and obtained a value of 0.56 from the initial source colonies to F1 and 0.57 for the F2 to F3 generation. These values are consistent with previous calculations of narrow sense heritability for hygienic behavior, which range from 0.36–0.65^15, 23, 25^. Our calculated values reinforce the high heritability of hygienic behavior in honey bees and show that that additive genetic variation contributes to the expression of this phenotype.

Hygienic behavior can confer resistance to brood pests and pathogens that contribute to honey bee colony losses. We therefore evaluated how well colonies headed by selected queens performed under disease (AFB) and *Varroa* mite challenge conditions. Selected and benchmark stocks, as well as an imported stock commonly used by Canadian beekeepers, were inoculated with either *Varroa* mites or *Paenibacillus larvae* spores at levels that would normally result in high levels of colony mortality.

**Figure 3:**
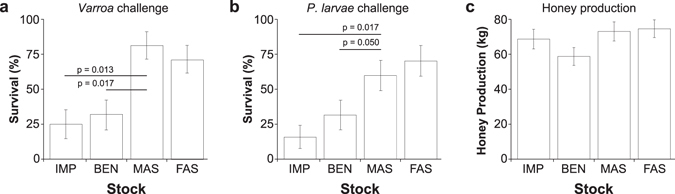
Performance of selected stock. IMP = imported stock, BEN = benchmark, MAS = Marker-assisted selection, FAS = Field-assisted selection. (**a**) Difference in winter survival of F3 generation colonies headed by queens from each stock type that were challenged with *Varroa* mites (*Varroa* challenge) (d.f. 3, Chi Sq 14.84 p > chi = 0.002). (**b**) Difference in symptom-free survival for colonies challenged with American foulbrood (*P. larvae*; AFB challenge) (d.f. 3, Chi Sq 12.65 p > chi = 0.0054). Horizontal lines represent Holm-Bonferonni adjusted single degree of freedom contrasts between MAS selected stock and the benchmark and imported stock controls. Similar results were found for FAS, with FAS survival higher than the BEN and IMP stocks for both the *Varroa* challenge (p = 0.05 and p = 0.025, respectively) and AFB challenge experiments (p = 0.025 and p = 0.013, respectively). Error bars represent the standard error of the binomial proportion. (**c**) Honey produced per colony for all stocks tested at three experimental sites in Alberta and Manitoba. There was no significant difference in honey production among the four stocks tested (d.f. 3,161; F = 2.12, p = 0.099).

To test for the ability to survive with *Varroa* mites, in the early summer 2013 we pooled a large population of worker bees from colonies that were infested with *V. destructor* (about three hundred mites per colony, or a 3.5% phoretic infestation rate based upon mites per 100 bees) and aliquoted 8,600 bees (1 kg of bees) into individual colonies. Twenty-three F3 selected or unselected queens were then randomly introduced into these individual colonies and the colonies were left untreated until the following spring; a fall survey for mites measured infestation rates of 23.0 ± 1.4 mites per 100 bees. For AFB, F3 colonies normalized for population received a frame with 225 cm^2^ of brood comb with 30 to 54% of brood cells showing clinical symptoms of AFB on each side. We assessed the impact of the parasitic mite by rating winter survival for colonies challenged with *Varroa*, and for colonies infected with *P. larvae*, and assessed overall survival as well as the presence of clinical symptoms of AFB (Fig. 3).

To verify that an independent but critical performance indicator of these stocks was not being degraded by too much focused selection on another trait, we also examined the ability of F3 colonies to collect honey in a separate study from the disease-challenge experiments. Reassuringly, honey production remained unaffected by selection for hygienic behavior, regardless of which selection method was used (Fig. 3c).

Our results clearly demonstrate that colonies founded by queens selected for hygienic behavior using MAS or FAS exhibited greater survival than control colonies in response to being challenged with *Varroa* or AFB. Therefore, using the freeze-killed brood assay or the marker selection panel, we were able to make gains in *Varroa* and AFB resistance and/or tolerance. However, with respect to *Varroa*, different assays or markers may select for distinct mechanisms of mite removal. Additional work is required to tease apart the relationship between Varroa Sensitive Hygiene and hygienic behavior as evaluated using the freeze-killed brood assay^13^. Eventually, markers could be tailored to discover colonies with either or both of these behavioral traits.

These results were subsequently used to model the economic impact of integrating the use of marker-selected stock into a Canadian beekeeping operation^26^. We presented a beekeeping case study where a beekeeper’s profit function was used to evaluate the economic impact of adopting colonies selected for hygienic behavior using MAS into an apiary. Our results showed a net profit gain from a MAS colony of between 2% and 5% when *Varroa* mites were effectively treated. In the case of ineffective treatment, MAS generated a net profit benefit of between 9% and 96% depending on the *Varroa* load. When a *Varroa* mite population had developed some treatment resistance, we showed that MAS colonies generated a net profit gain of between 8% and 112% depending on the *Varroa* load and degree of treatment resistance.

Though the first transgenic modification of bees has been reported^27^, it is unlikely that industrial use of genetically-modified honey bees will be accepted by the public at this time. Therefore, tools that enable accelerated stock enrichment without resorting to genetic modification are highly desirable. While genetic markers for hygienic behavior and *Varroa*-sensitive hygiene have been identified^16, 17^, the existing ones are unlikely to be tightly enough linked to the causal genes to be robust to the high recombination rate that honey bees exhibit. These challenges are likely to be overcome with higher densities of markers or genome-wide association studies. Expression markers integrate many different *cis-* (e.g., transcriptional enhancer elements) and *trans-* (e.g., transcription factors) effects so if they are functionally linked to the trait in question then they should be robust to recombination. We have not yet shown that the markers we use here are functionally linked to FKB removal, *Varroa*-sensitive hygiene, or grooming behavior but they are tightly linked enough to enrich the trait as quickly as the best conventional methods available.

Selective breeding is a vital tool for improving yields and disease resistance in all plants and animals used in agriculture. MAS has the potential to be more precise and more robust to external influences; it has been widely used in certain plants^28^ and animals^29^. To date, however, the markers used have been genomic loci exclusively, starting with restriction fragment length polymorphisms followed by single nucleotide polymorphisms and whole genome profiling. This is undoubtedly due, in part, to the availability of efficient genetic approaches for finding such markers. It is also a matter of focus: researchers have spent more time looking for genetic loci than for expression markers (i.e., transcripts or proteins) because the latter have previously been considered to be too dependent on environment. Here we have shown that expression markers can be used to select for a very complex, polygenic trait. Even in this proof-of-principle with a first-generation panel of markers, MAS was as efficient at enriching disease-resistance as FAS methods (Fig. 3a and b): bees bred using marker-assisted selection could resist levels of disease that would typically kill 70% or more of unselected colonies. The data presented here have implications beyond bees: this is the first demonstration of marker-assisted selection in livestock using expression markers and it enables molecular diagnostic approaches for selecting complex polygenic traits that are recalcitrant to genetic mapping methods^30^.

## Methods

### Queen stocks

The stocks used in this study were: (1) Field-assisted selection (FAS): F1 and F3 queens were selectively bred using the standard hygienic behavior assay described above, (2) Marker-assisted selection (MAS): F1 and F3 queens were selectively bred based on protein markers associated with hygienic behavior, and (3) Benchmark (BEN): F1 and F3 queens reared and open-mated in British Columbia in the spring of 2012/13 from stocks randomly selected from beekeepers across western Canada in 2011, or (4) Import (IMP): Commercially reared and open-mated queens purchased from New Zealand suppliers in May 2013. All experimental queens were paint-marked, with one wing clipped to enable their identification. Any colonies with new queens produced by swarming or supersedure were removed from subsequent evaluations, as were all queenless colonies. All F1 and F3 queens for evaluation were shipped to common sites and tested at the same time and location.

### Reagents

All chemicals used were of analytical grade or better and all solvents were of HPLC-grade or better; all, with the exceptions specified below, were obtained from ThermoFisher-Scientific (St. Waltham, MA, USA): porcine modified trypsin (Promega, Nepean, Ontario, Canada); 96-well full skirt PCR plates (Axygen, Union City, CA, USA); stable isotope-labelled standard peptides were synthesized using Fmoc chemistry on a Prelude peptide synthesizer (Protein Technologies, Inc., Tuscon, AZ).

### Ethics statement

As honey bees are an uncontrolled (i.e., non-cephalopod) invertebrate, the University of British Columbia’s Animal Care Committee did not require specific ethics certification for this research.

### Bee sampling, hygienic behavior testing and beekeeping

Prior to each hygienic test, the queen status of each colony was assessed. Only queenright colonies were evaluated, and for experimental colonies and the selection program, the marked experimental queen was located prior to testing. A suitable brood frame with a solid patch of capped brood was then identified and marked for each colony. The test adapted methods described by Spivak *et al*.^12^: specifically, two polyvinyl chloride (PVC) pipes (6 cm outer diameter and 5 cm inner diameter, cut 15 cm long) were pressed and slightly twisted into the brood comb. Liquid nitrogen was used to freeze the brood and the number of empty brood cells or cells with pollen/honey in each of the two resultant freeze-killed circles was recorded and subtracted from the total number of cells in the patch, to determine the initial number of capped brood cells frozen per patch. Each frame was then re-inserted into the center of the brood nest of the colony it came from. After 24 h, the number of capped and partially removed cells for each of the two freeze-killed brood circles on each frame was recorded and used to calculate the percentage of brood cells removed. The number of completely removed cells were used in calculation of hygienic score. The test was repeated one week from the first freeze, for a total of two sets of two freeze-killed brood patches (i.e., four patches total). The score from the two testing dates were averaged to produce the colony hygienic behavior score.

For the initial survey, 635 colonies in 38 commercial beekeeping operations across British Columbia, Alberta, and Manitoba were tested for hygienic behavior, defined here as proportion of freeze-killed brood completely removed within 24 h. A sample of ~50 bees from the brood nest was also collected for marker profiling. Queens were then moved to one of four sites: those to be used for selective breeding were shipped either to apiaries near Grand Forks, BC (49°N, 118°W) or Langley, BC (49°N, 122°W) while those comprising the benchmark populations were moved either to Langley, BC, the Agriculture and Agri-Food Canada Research Farm in Beaverlodge, AB (55°N, 119°W) or the University of Manitoba (50°N, 97°W). The populations were then maintained at these locations and queens were shipped via air freight to the experimental sites, as needed. All results reported on selected lines, BEN, and IMP include only comparisons made at the same site at the same time (i.e. we only compare hygienic behavior, *Varroa* levels, and survival for BEN, IMP, MAS, and FAS when evaluations were conducted for all lines at the same site at the same time).

### Protein extraction from antennae

Pooled antennae from at least 30 bees per colony were washed three times with phosphate-buffered saline (PBS) and bead-homogenized in buffer (50 mM Tris-Cl, 150 mM NaCl, 1% NP-40, 1% DTT) for three 20 s bursts at 6.5 M/s, with 1 min rest on ice between each burst. Insoluble material was pelleted at 600 relative centrifugal force (RCF) and protein was precipitated from the supernatants using 1000 µL of ethanol, 25 µL of 2.5 M sodium acetate (pH 5.5) and 5 µL of glycogen (10 mg/ml). The precipitation was allowed to proceed at room temperature for 120 min. After centrifugation twice at 16,000 r.c.f. for 15 min, the pellets were dried and resuspended in solubilization buffer (50 mM ammonium bicarbonate, 1% sodium deoxycholate) at 99 °C for 5 min. The samples were then sonicated in water bath for 5 min and any insoluble material was removed by centrifugation at 16,000 r.c.f. for 15 min. Protein concentrations were measured by a BCA protein assay using serial dilutions of bovine serum albumin to generate a standard curve. For each sample, 20 µg of protein was diluted to 0.8 µg/µl in solubilization buffer and sent to the University of Victoria Genome BC Proteomics Centre.

### MRM analysis

Sample manipulations for in-solution digestion, SIS peptide addition, and SPE cleanup steps were performed using a Tecan Freedom Evo150 liquid-handling robot. Samples were diluted to 0.4 µg/µL in solubilization buffer, denatured by adding 20 µL of 4.5% w/v sodium deoxycholate, reduced by adding 20 µL of 5 mM tris(2-carboxyethyl) phosphine in 50 mM ammonium bicarbonate, and incubating at 60 °C for 30 min. Free sulfhydryl groups were alkylated by adding 20 µL of 20 mM iodoacetamide (in 50 mM ammonium bicarbonate) and incubating at 37 °C for 30 min. Remaining iodoacetamide was quenched by adding of 20 µL of 20 mM dithiothreitol (in 50 mM ammonium bicarbonate) and incubating at 37 °C for 30 min. Samples were digested by adding 10 µL of trypsin (0.1 µg/µL in 50 mM ammonium bicarbonate) and incubating at 37 °C for 16 h. Digestion was stopped by adding 20 µL of a stable-isotope-labeled standard (SIS) peptide mixture and 20 uL of 4.5% v/v formic acid. Samples were centrifuged 10 min at 3,000 r.c.f (23 °C) and 13 µg of digest was desalted and concentrated by solid phase extraction using Waters Oasis HLB mElution plate (2 mg resin). Wash was 0.5 mL water, and eluent was 75 µL of 50% acetonitrile, 0.1% formic acid. Eluted samples were frozen and lyophilized to dryness overnight. Samples were rehydrated in Solvent A (0.1% v/v formic acid) for LC-MRM/MS analysis.

LC-MRM/MS analysis was done using an Agilent 6490 Triple Quad LC/MS coupled to an Agilent 1290 Infinity UHPLC. The analytical column was 2.1 × 150 mm Agilent ZORBAX Eclipse Plus C18 Rapid Resolution HD column, 1.8 μm particles at column temperature of 50 °C. Ten µg of sample were directly loaded onto the analytical column at 0.4 mL/min with 3% solvent B (90% v/v acetonitrile, 0.1% v/v formic acid). Samples were separated using a flow rate of 0.4 mL/min with a 2 min linear gradient from 3 to 13% solvent B, then a 15 min linear gradient from 13 to 20% solvent B, followed by a 6 min linear gradient from 20 to 27% solvent B, then a 3 min linear gradient from 27 to 44% solvent B, and finally a 2 min linear gradient from 44 to 90% solvent B.

All acquisition methods used the following parameters: 3500 V capillary voltage, a sheath gas flow of 11 L/min. (UHP nitrogen), a 200 °C sheath gas temperature, an MS operating pressure of 5.08 × 10-5 Torr, and Q1 and Q3 set to unit resolution. MRM acquisition methods were constructed using 3 ion pairs per peptide with fragment ion specific tuned CE voltages and retention time constraints. A default 380 V fragmentor voltage and 5 V cell accelerator potential were used for all MRM ion pairs, and the dynamic-MRM option was used for all data acquisition with a target cycle time of 1 second and a 0.9 min MRM detection window.

All MRM data was processed using Agilent MassHunter Quantitative Analysis (Agilent B.04.00) with the Agile Integrator algorithm for peak integration set with default values. Peak images of extracted ion chromatograms of each transition in all peptides and samples were retrieved and manually reviewed for inconsistent and/or poor signals. For F1 and F2 datasets, signals which the software could not integrate automatically or that generated poor peak identification due to low signal-to-noise ratio were assigned a limit-of-detection value (LOD: half of lowest ratio observed for whole data set). Reported Relative Response (RR) is the ratio of the integrated area of the endogenous (natural) peak to the integrated area of the corresponding standard (SIS) peptide. RR are derived from the quantifier MRM transition, with two qualifier transitions acting to verify retention times and reveal any signal interference.

### Application of biomarker panels

For breeding the F1 and F2 generations, the parents were ranked according to the probability of having a high hygienic behavior values as estimated using their MRM peptide levels and the predictive panel for that generation (Supplemental Table 2). The optimal panel was re-optimized during each generation based on the correlation between hygienic behavior scores and marker levels in the FAS colonies.

### Instrumental insemination and closed mating

For F1 and F2 generations, selected virgin queens were instrumentally inseminated^8^ at each of two sites, Grand Forks and Langley, British Columbia, using semen subsampled from a pooled sample from drones collected from 8–12 breeder colonies per stock (FAS or MAS) per site. Virgin queens in the F1 generation were inseminated with 4 μl semen as fewer mature drones were available at the early spring insemination date (May 9–12, 2012), whereas queens in the F2 generation were inseminated with 8 μl of semen from the appropriate selected stock later in the summer when more mature drones were available (August 8–15, 2012). A total of 153 MAS and 159 FAS queens were inseminated, with 83 MAS and 73 FAS tested as potential breeders for the F2 generation, and the remaining successfully laying queens sent to other sites for experimental field trials. A total of 70 F2 FAS and 69 F2 MAS were tested for hygienic behavior at the breeding sites for potential inclusion in the pool of breeder colonies used to breed the F3 generation. F3 queens were closed mated because the large number of queens required for experiments rendered instrumental insemination prohibitively difficult. The F3 mating apiaries were located 19 km apart from each other (49° 0′ 22.47″ N, 118° 31′ 4.32″ W and 49° 1′ 2.01″ N, 118° 13′ 34.0″ W) in the proximity of Grand Forks, BC, and Christina Lake, BC, respectively, where there were no other drone sources at the time of mating. Virgin queens were produced from the top ranked 9 MAS and 10 FAS colonies, and an additional 9 MAS and 10 FAS colonies were used only as drone sources in the isolated mating yards. The F3 queens were allowed to emerge and mate naturally with the selected drones and the colonies were inspected after 7 and 10 d to identify successfully-mated queens.

### Analysis of Hygienic Behavior and Realized Heritability

The hygienic behavior data analysis was generated using SAS software (SAS, V. 8.0, SAS Institute, Cary, NC). F0 data were analysed by ANOVA using the general linear model procedure following arcsine transformation of the proportion of cells removed. The F-test for comparisons among provinces was significant (d.f. 2, 592; F = 16.03, P = 0.0001) comparison of means was done using Tukey’s multiple range test. Comparison of the subset of colonies selected as the benchmark population with the overall mean of all colonies from all provinces was done using an analysis of means procedure (Proc Glm, Pdiff = ANOM) (d.f. 1, 593; F = 1.55, P = 0.2134). For comparisons of progress in selection for both the F1 and F3 generations data were first compared using a two-way ANOVA using Proc Mixed (REML), with generation and genetic line as factors and locality (apiary location) as a blocking factor. Because a significant interaction occurred between generation and genetic line (d.f. 2, 267; F = 3.09, P = 0.047), single degree of freedom contrasts were made within stocks using the slice command. Comparisons of stocks within each generation were carried out using Tukey’s multiple range test.

Realized heritability was calculated according to recognized methods^31^.

$$\begin{array}{rcl}{\rm{Realized}}\,{\rm{heritability}} & = & {\rm{Response}}\,{\rm{to}}\,{\rm{selection}}/{\rm{Selection}}\,{\rm{differential}}\\ {\rm{Realized}}\,{\rm{Heritability}} & = & \frac{{\rm{Response}}\,{\rm{to}}\,{\rm{selection}}}{{\rm{Selection}}\,{\rm{differential}}}\\  & = & \frac{{\rm{average}}\,{\rm{1st}}\,\mathrm{generation}-\mathrm{average}\,{\rm{2nd}}\,{\rm{generation}}}{{\rm{average}}\,{\rm{1st}}\,\mathrm{generation}-\mathrm{average}\,{\rm{selected}}\,{\rm{breeders}}}\end{array}$$

Where in the first breeding cycle: the ‘1^st^ generation’ was the initial source colonies, the ‘2^nd^ generation’ was F1, and the selected breeders were the selected F0 parents. For the F2 to F3: the ‘1st generation’ was F2, the ‘2nd generation’ was F3, and the selected breeders were the selected F2 parents. All values of F1, F2 and F3 used in these calculations were obtained from the BC breeder sites.

### American Foulbrood Challenge experiment

Large, double brood chamber colonies from Agriculture and Agri-Food Canada’s Beaverlodge Research Farm were divided on 24 May 2013 to produce small experimental colonies (splits) in which to introduce the experimental queens. Each split consisted of three frames of bees and brood, two frames with stored honey and pollen, and four frames of undrawn plastic (foundation) on which the bees could build new comb. The colonies were divided evenly between two apiaries. Once all colonies were established with laying queens, and worker populations had turned over so that the colony consisted of progeny from the experimental queen (8 weeks) an initial hygienic behavior assay was performed.

Frames with AFB were donated by commercial beekeepers in Alberta, and 15 × 15 cm sections of comb were cut from the most heavily infected of these frames. In each section, 24–53% of cells had clinical symptoms of AFB (sunken and perforated cappings, ropey larvae, or scale) on both sides of each section, as determined by visual inspection. The cut sections were then randomly assigned to a colony and placed in a similarly-sized hole cut from brood frames that showed no visible signs of disease. These frames were placed in the centre of the brood nests of the experimental colonies in July 2013 (F3).

During the fall of 2013, all colonies were fed fumagillin-medicated syrup (Fumagilin-B®) to suppress *Nosema* spp. infections. On 31 October 2013, colonies were wintered indoors at Beaverlodge Research Farm under conditions of constant darkness and standard ventilation at 5 °C^28^. Asymptomatic colonies (free from any visual signs of disease) were considered to have survived from the period after initial acceptance until the following spring if they contained the experimental queen and any number of workers on 9–12 May 2014. The proportion of asymptomatic colonies surviving (pooled over both apiaries) was analysed using logistic regression (Proc Catmod, SAS) and where the overall model was significant, one-tailed single degree of freedom contrasts were carried out to test whether survival of each selected stock (MAS or FAS) was improved relative to each benchmark (IMP or BEN).

### *Varroa destructor* challenge experiment

The F3 generation of queens, benchmark and imported stock were also assessed to quantify the effects of inoculation with *Varroa destructor* on winter survival of colonies. Ninety-two colonies of *Varroa*-infested European honey bees were established in two apiary sites on the University of Manitoba campus, Winnipeg, Manitoba, Canada in June of 2013 (44 colonies were placed in one site and 48 in another located 2.7 km away from the first). Colonies consisting of 1 kg (about 8,600 bees) of worker bees each infested with approximately 300 *Varroa* mites (i.e., 3.5% infestation), were established in single brood chambers containing nine frames of foundation and one frame of honey.

Uniform starting populations of bees and *Varroa* mites were obtained by combining mite- infested bees from several hives into large screened boxes (133 by 72 by 68 cm). Source colonies consisted of 30 *Varroa*-infested colonies from the University of Manitoba Apiary, 50 colonies that originated from Australian packages, and 30 colonies of *Varroa*-infested bees obtained from a local producer. On 5 June, frames of bees were shaken from the University of Manitoba colonies (25.7 kg) and Australian packages (70.1 kg) into two large cages. These were maintained in a cool dark room overnight to allow thorough mixing of the bees and mites from different source colonies. Six sample cups of workers were taken from different locations in each cage (a total of 4,941 bees). The following morning, these were weighed, counted and subjected to an alcohol wash, to obtain an average weight per bee and to determine the number of mites per gram of bees for each cage. A mixture of 450 g of mites and bees was then shaken into each brood chamber with entrances screened, and an experimental queen was added (in a cage). The inoculated colonies with queens were then left overnight to settle before moving them out to the experimental yards, releasing the queens and opening the hive entrances. In order to obtain the desired target starting populations of bees and mites, a second inoculation was carried out one week later. Bees purchased from a local producer that had not been treated with acaricide were shaken into a single large cage (51.8 kg of bees), sampled 14 times (a total of 4880 bees) and processed as described previously. An additional allocation of bees and mites were added to each colony to bring the starting population to 300 mites and 1 kg of bees. These bees were added to existing colonies by pouring them into an empty hive top feeder that was placed on top of each colony with a piece of newspaper placed between the brood chamber and feeder. This was done to unite the additional bees to the colony in such a way as to minimize loss of the project queens.

Colonies were spaced about 1 m apart with entrances facing in different directions to minimize drift between colonies. Upon establishment colonies were immediately fed sugar syrup (2 part sucrose to 1 part water) using a hivetop feeder and each received a 0.5 kg 15% pollen patty. Each of the four queen stock treatments (described above) was randomly assigned to colonies. Caged queens were introduced into colonies and colonies were maintained as single brood-chamber units throughout the summer with boxes for honey storage being added above a queen excluder as required.

Fall management was performed according to normal commercial practice for the region, with the exception of colonies receiving treatment for *Varroa* mites. All colonies were fed with fumagillin-medicated syrup (Fumagilin-B^®^) and received oxytetracycline (Oxytet-25^®^) treatments in powdered icing sugar, according to label directions. Prior to wintering, colonies were sampled using an alcohol wash to assess phoretic *Varroa* infestation levels and then were wintered indoors at the University of Manitoba campus on 31 October 2013. Colonies were maintained under constant darkness and standard ventilation^32^ at 5 °C. Winter survival of colonies was assessed when the colonies were removed from the wintering building on 17 April 2014. Colonies containing the experimental queen and any number of workers were considered to be alive. Overwintering survival was calculated based upon the number of live colonies in spring (any number of bees with a live queen) divided by the number of live colonies in late fall. Data were analysed as described above for overall survival of asymptomatic colonies in the AFB challenge experiment.

### Honey Production Assessment

Packaged bees (1 kg) imported from New Zealand were hived at three locations: Lethbridge, Alberta (48 colonies, 1 apiary), Beaverlodge, Alberta (96 colonies, 2 apiaries), and Glenlea, Manitoba (96 colonies, 2 apiaries) in May 2013. Original queens were removed from all packages and experimental queens were then introduced into each queenless package. Only colonies with laying experimental queens were included in the experiment. Colonies headed by queens of each of the four experimental stocks (MAS, FAS, BEN, and IMP) were distributed throughout the apiary and blocked in groups of four with one colony of each stock type randomly positioned within each block. Colony entrances at each site were rotated in such a way that colonies from each stock type faced different directions. Queens were confined to single brood-chamber hives, maintained following commercial management practices, and supplied with pre-weighed honey supers (boxes for harvesting honey), as required. Half of the colonies within each stock were randomly assigned treatment with amitraz (Apivar^®^), according to label instructions; nevertheless, this mite treatment had no measurable effect on honey yield as mites were intentionally at very low levels in the source colonies and therefore was not included as a factor in the analysis. The total amount of honey produced was evaluated for a total of 191 experimental colonies across the five sites, and only colonies that retained their experimental queen for the duration of the summer were included in the honey production analyses (n = 169). Total honey production per colony was determined by weighing filled honey supers and subtracting their empty weight. Differences among queen stocks were analysed by ANOVA (Proc Mixed, REML, SAS) using apiary site as a blocking factor. Data were log-transformed prior to analysis.

### Significance statement

The plight of the honey bee has received world-wide attention in recent years, principally because of greater than normal levels of mortality recorded from across the globe, resulting from many pressures on bee health. Among these are a milieu of pathogens and parasites, including the parasitic mite *Varroa destructor*, which has become resistant to a succession of acaricides used for its control by beekeepers. In this study, we show that robust expression biomarkers of a disease-resistance trait can be used to select for that trait. After three generations of selection, the resulting stock performed significantly better than an unselected benchmark population when challenged with disease or *Varroa* mites. This is the first demonstration of an expression marker for selective breeding in any agricultural species, plant or animal. This also represents a completely novel way to select for highly polygenic traits.

## Electronic supplementary material

Supplementary information

Supplementary Table 1

Dataset 2

Supplementary table 2
