# Supplementary material for: Peptide biomarkers used for the selective breeding of a complex polygenic trait in honey bees
Source: Sci Rep. 2017 Aug 21;7:8381. doi: 10.1038/s41598-017-08464-2 (PMC5566959; doi:10.1038/s41598-017-08464-2)
Supplement: Supplementary file 1 — Supplementary information [file 41598_2017_8464_MOESM1_ESM.pdf]

Supplemental materials for:

Peptide biomarkers used for the selective breeding of a complex polygenic trait in honey bees

M. Marta Guarna[#,1,2+], Shelley E. Hoover[#,1,2, 3+], Elizabeth Huxter[4], Heather Higo[1], Kyung-Mee Moon[1], Dominik Domanski[5], Miriam E.F. Bixby[1], Andony P. Melathopoulos[2,3,6+], Abdullah Ibrahim[2], Michael Peirson[2], Suresh Desai[7], Derek Micholson[7], Rick White[8], Christoph H. Borchers[5], Robert W. Currie[7], Stephen F. Pernal[2]*, Leonard J. Foster[1]*

[1]Department of Biochemistry & Molecular Biology, and Centre for Sustainable Food Systems, University of British Columbia, Vancouver, BC, Canada,

[2]Agriculture & Agri-Food Canada, Beaverlodge Research Farm, Beaverlodge AB, Canada

[3]Alberta Agriculture and Forestry, Lethbridge, AB, Canada

[4]Kettle Valley Queens, Grand Forks, BC, Canada

[5]Genome BC Proteomics Centre, University of Victoria, Victoria, BC, Canada

[6]Department of Biological Sciences, University of Calgary, AB, Canada

[7]Department of Entomology, University of Manitoba, Winnipeg, MB, Canada

[8]Department of Statistics, University of British Columbia, Vancouver, BC, Canada

[#]These authors contributed equally to this work

[+]Current affiliation

*Address correspondence to:

LJF - foster@chibi.ubc.camailto:foster@chibi.ubc.ca

SFP - Steve.Pernal@agr.gc.ca

Supplementary Tables

Supplementary Table 1 – Peptides and MRM transitions

Peptides from each protein linked to the different behaviours are listed, along with the MRM transitions used for their detection.

Supplementary Table 2 – MRM values from all colonies

Four spreadsheets are included representing each of the four generations, including F0. Data are relative ratios for the 1° MRM transition for each peptide versus the stable isotope-labelled standard. The peptide codes in the first row are coded by HB or CTRL (for marker or control), the protein name (corresponding to those in Supplementary Table 1) and the first two letters of the amino acid sequence (corresponding to those in Supplementary Table 1). The colony number, province it was located and %HB scores are also given.

Supplementary Table 3 – Peptide panels

The top-performing peptide MRM panels from F2 and F3.

**Panel F2**	**Panel F3**
HB-OBP18-EI	HB-TaTBP-DS
Gr-ASP3-LL	Gr-ASP3-FD
HB-OBP18-FN	HB-CoAT-LG
HB-P450-AE	HB-CoAT-LP
HB-P450-DL	HB-Hyp-HT
HB-P450-LS	HB-Hyp-MG
HB-VAMP-ED	HB-OBP18-EI
HB-VAMP-ED:HB-OBP18-FN	HB-VAMP-EI
	HB-VAMP-FR
	HB-VAMP-TV
	VSH-VAT1-GA

On-line Methods

Reagents

All chemicals used were of analytical grade or better and all solvents were of HPLC-grade or better; all, with the exceptions specified below, were obtained from ThermoFisher-Scientific (St. Waltham, MA, USA): porcine modified trypsin (Promega, Nepean, Ontario, Canada); 96-well full skirt PCR plates (Axygen, Union City, CA, USA); stable isotope-labelled standard peptides were synthesized using Fmoc chemistry on a Prelude peptide synthesizer (Protein Technologies, Inc., Tuscon, AZ), as described[1].

Ethics statement

As an uncontrolled (i.e., non-cephalopod) invertebrate, the University of British Columbia's Animal Care Committee does not require specific ethics certification for honey bees.

Bee sampling, hygienic behaviour testing and beekeeping

Prior to each hygienic test, the queen status of each colony was assessed. Only queenright colonies were tested, and for experimental colonies and the selection program, the marked experimental queen was located prior to testing. A suitable brood frame with a solid patch of capped

brood was then identified and marked for each colony. The test followed methods described by Spivak et col.[2]: two polyvinyl chloride (PVC) pipes (6 cm outer diameter and 5 cm inner diameter, cut 15 cm long) were pressed and slightly twisted into the brood comb. Liquid nitrogen was used to freeze the brood and the number of empty brood cells or cells with pollen/honey in each of the two resultant freeze-killed circles was recorded and subtracted from the total number of cells in the patch, to determine the initial number of capped brood cells frozen per patch. Each frame was then re-inserted into the center of the brood nest of the colony it came from. After 24 h, the number of capped and partially removed cells for each of the two freeze-killed brood circles on each frame was recorded and used to calculate the percentage of brood cells removed. The number of completely removed cells were used in calculation of hygienic score. The test was repeated one week from the first freeze, for a total of two sets of two freeze-killed brood patches (i.e., four patches total). The score from the two testing dates were averaged to produce the colony hygienic behaviour score.

For the initial survey, 635 colonies in 38 commercial beekeeping operations across British Columbia, Alberta, and Manitoba were tested for hygienic behaviour, defined here as proportion of freeze-killed brood completely removed within 24 h. A sample of ~50 bees from the brood nest was also collected for marker profiling. Queens were then moved to one of four sites: those to be used for selective breeding were moved either to apiaries near Grand Forks, BC (49˚N, 118˚W) or Langley, BC (49˚N, 122˚W) while those comprising the benchmark populations were moved either to Langley, BC, or the Agriculture and Agri-Food Canada Beaverlodge Research Farm, AB (55˚N, 119˚W) or the University of Manitoba (50˚N, 97˚W). The populations were then maintained at these locations and queens were shipped via air freight to the experimental sites, as needed.

Protein extraction from antennae

Pooled antennae from at least 30 bees per colony were washed three times with phosphate-buffered saline (PBS) and bead-homogenized in buffer (50 mM Tris-Cl, 150 mM NaCl, 1 % NP-40, 1 % DTT) for three 20 s bursts at 6.5 M/s, with 1 min rest on ice between each burst. Insoluble material was pelleted at 600 relative centrifugal force (RCF) and protein was precipitated from the supernatants using 1000 µL of ethanol, 25 µL of 2.5 M sodium acetate (pH 5.5) and 5 µL of glycogen (10 mg/ml). The precipitation was allowed to proceed at room temperature for 120 min. After centrifugation twice at 16,000 r.c.f. for 15 min, the pellets were dried and solubilized in solubilization buffer (50 mM ammonium bicarbonate, 1 % sodium deoxycholate) at 99°C for 5 min. The samples were then sonicated in water bath for 5 min and any insoluble material was removed by centrifugation at 16,000 r.c.f. for 15 min. Protein concentrations were measured by a BCA protein assay using serial dilutions of bovine serum albumin to generate a standard curve. For each sample, 20 µg of protein was diluted to 0.8 µg/µl in solubilization buffer and sent to the University of Victoria Genome BC Proteomics Centre.

MRM analysis:

Sample manipulations for in-solution digestion, SIS peptide addition, and SPE cleanup steps were performed using a Tecan Freedom Evo150 liquid-handling robot. Samples were diluted to 0.4 µg/µL in solubilization buffer, denatured by adding 20 µL of 4.5% w/v sodium deoxycholate, reduced by adding 20 µL of 5 mM tris(2-carboxyethyl) phosphine in 50 mM ammonium bicarbonate, and incubating at 60°C for 30 min. Free sulfhydryl groups were alkylated by adding 20 µL of 20 mM iodoacetamide (in 50 mM ammonium bicarbonate) and incubating at 37°C for 30 min. Remaining iodoacetamide was quenched by adding of 20 µL of 20 mM dithiothreitol (in 50 mM ammonium bicarbonate) and incubating at 37°C for 30 min. Samples were digested by adding 10 µL of trypsin

(0.1 μg/μL in 50 mM ammonium bicarbonate) and incubating at 37°C for 16 hours. Digestion was stopped by adding 20 μL of a stable-isotope-labeled standard (SIS) peptide mixture and 20 uL of 4.5% v/v formic acid. Samples were centrifuged 10 min at 3,000 × g (23°C) and 13 μg of digest was desalted and concentrated by solid phase extraction using Waters Oasis HLB mElution plate (2 mg resin). Wash was 0.5 mL water, and eluent was 75 μL of 50% acetonitrile, 0.1% formic acid. Eluted samples were frozen and lyophilized to dryness overnight. Samples were rehydrated in Solvent A (0.1% v/v formic acid) for LC-MRM/MS analysis.

LC-MRM/MS analysis was done using an Agilent 6490 Triple Quad LC/MS coupled to an Agilent 1290 Infinity UHPLC. The analytical column was 2.1x 150 mm Agilent ZORBAX Eclipse Plus C18 Rapid Resolution HD column, 1.8 μm particles at column temperature of 50˚C. Ten μg of sample were directly loaded onto the analytical column at 0.4 mL/min with 3% solvent B (90% v/v acetonitrile, 0.1% v/v formic acid). Samples were separated using a flow rate of 0.4 mL/min with a 2 min linear gradient from 3 to 13% solvent B, then a 15 min linear gradient from 13 to 20% solvent B, followed by a 6 min linear gradient from 20 to 27% solvent B, then a 3 min linear gradient from 27 to 44% solvent B, and finally a 2 min linear gradient from 44 to 90% solvent B.

All acquisition methods used the following parameters: 3500 V capillary voltage, a sheath gas flow of 11 L/min. (UHP nitrogen), a 200°C sheath gas temperature, an MS operating pressure of 5.08 × 10-5 Torr, and Q1 and Q3 set to unit resolution. MRM acquisition methods were constructed using 3 ion pairs per peptide with fragment ion specific tuned CE voltages and retention time constraints. A default 380 V fragmentor voltage and 5 V cell accelerator potential were used for all MRM ion pairs, and the dynamic-MRM option was used for all data acquisition with a target cycle time of 1 second and a 0.9 min MRM detection window.

All MRM data was processed using Agilent MassHunter Quantitative Analysis (Agilent B.04.00) with the Agile Integrator algorithm for peak integration set with default values. Peak images of extracted ion chromatograms of each transition in all peptides and samples were retrieved and manually reviewed for inconsistent and/or poor signals. For F1 and F2 datasets, signals which the software could not integrate automatically or that generated poor peak identification due to low signal-to-noise ratio were assigned a limit-of-detection value (LOD: half of lowest ratio observed for whole data set). Reported Relative Response (RR) is the ratio of the integrated area of the endogenous (natural) peak to the integrated area of the corresponding standard (SIS) peptide. RR are derived from the quantifier MRM transition, with two qualifier transitions acting to verify retention times and reveal any signal interference.

Application of biomarker panels

For breeding the F1 and F2 generations, the parents were ranked according to the probability of having a high hygienic behavior values as estimated using their MRM peptide levels and the predictive panel for that generation (Supplemental Table 2). The optimal panel was re-optimized during each generation based on the correlation between HB scores and marker levels in the FAS colonies.

Instrumental insemination and closed mating

For F1 and F2 generations, selected virgin queens were instrumentally inseminated[3] using a mix of semen collected from at least 20 mature drones from the appropriate selected stock. F3 queens were naturally mated because of the number of queens required for experiments made instrumental insemination impossible. The F3 mating apiaries were located in a remote mountain valley north of

Grand Forks, BC, where there are no other known feral or domestic honey bees. The queens were allowed to emerge and mate naturally with the selected drones and the colonies were inspected after 7 and 10 d to identify successfully mated queens.

Queen stocks

The stocks used in this study were: (1) Field-assisted selection (FAS): F1 and F3 queens were selectively bred using the standard hygienic behaviour assay described above, (2) Marker-assisted selection (MAS): F1 and F3 queens from a line selectively bred based on protein markers associated with hygienic behaviour, and (3) Benchmark (BEN): F1 and F3 queens reared and open-mated in British Columbia in the spring of 2012/13 from stocks randomly selected from beekeepers across western Canada in 2011, or (4) Import (IMP): Commercially reared and open-mated queens purchased from New Zealand suppliers in May 2013. All experimental queens were marked, with one wing clipped to enable their identification. Any colonies with new queens produced by swarming or supersedure were removed from subsequent evaluations, as were all queenless colonies.

Statistical Analysis of Hygienic Behaviour

F0 data were analysed by ANOVA using the general linear model procedure (SAS) following arcsine transformation of the proportion of cells removed. The F-test for comparisons of among provinces was significant (d.f. 2, 592; F=16.03, p=0.0001) comparison of means was done using Tukey's multiple range test. Comparison of the subset of colonies selected as the benchmark population with the overall mean of all colonies from all provinces was done using an analysis of means procedure (SAS) (d.f. 1, 593; F=1.55, p=0.2134). For comparisons of progress in selection for both the F1 and F3 generations data were first compared using a two way ANOVA using Proc Mixed (REML), with generation and genetic line as factors and locality (apiary location) as a blocking factor. Since a significant interaction occurred between generation and genetic line (d.f. 2, 267; F= 3.09, p=0.047), single degree of freedom contrasts were made within stocks using the slice command (SAS). Comparisons of stocks within each generation were carried out using Tukey's multiple range test.

American Foulbrood Challenge experiment

Large, double brood-box colonies from the Agriculture and Agri-Food Canada Beaverlodge Research Farm were divided on 24 May 2013 to produce small experimental colonies (splits) in which to introduce the experimental queens. Each split consisted of three frames of bees and brood, two frames with stored honey and pollen, and four frames of undrawn plastic (foundation) on which the bees could build new comb. The colonies were divided evenly between two apiaries. Once all colonies were established with laying queens, and worker populations had turned over so that the colony consisted of progeny from the experimental queen (8 weeks) an initial hygienic behaviour assay was performed.

Frames with AFB were donated by commercial beekeepers in Alberta, and 15 x 15 cm sections of comb were cut from the most heavily infected of these frames. In each section, 24-53% of cells had visible AFB symptoms (sunken and perforated cappings, ropey larvae, or scale), on both sides of each section, as determined by visual inspection. The cut sections were then randomly assigned to a colony and placed in a similarly-sized hole cut from brood frames that showed no visible signs of disease. These frames were placed in the centre of the brood nests of the experimental colonies in July 2013 (F3)[2].

All colonies were overwintered indoors at 5˚C[4] in the same facility at Beaverlodge Research Farm. All asymptomatic colonies (free from any visual signs of disease) were considered to have survived from the period after initial acceptance until the following spring if they contained the experimental queen and any number of workers on 9-12 May 2014. The proportion of asymptomatic colonies surviving (pooled over both apiaries) was analysed using logistic regression(Proc Catmod, SAS) and where the overall model was significant, one-tailed single degree of freedom contrasts were carried out to test whether survival of each selected stock (MAS or FAS) was improved relative to each benchmark (IMP or BEN).

***Varroa destructor* challenge experiment**

The F3 generation of queens, benchmark and imported stock were also assessed to quantify the effects of inoculation with *Varroa destructor*[5] on winter survival of colonies. In 2013, ninety two colonies of *Varroa*-infested European honey bees were established in two apiary sites on the University of Manitoba campus, Winnipeg, Manitoba, Canada in June of 2013 (44 colonies were placed in one site and 48 in another located 2.7km away from the first). Colonies consisting of 1 kg (about 8,600 bees) of worker bees each infested with approximately 300 *Varroa* mites (i.e., 3.3% infestation), were established in single brood chambers containing nine frames of foundation and one frame of honey. Colonies were spaced about 1 m apart with entrances facing in different directions to minimize drift between colonies. Upon establishment colonies were immediately fed sugar syrup (2 part sucrose to 1 part water) using a hivetop feeder and each received a 0.5 kg 15% pollen patty. Each of the four queen stock treatments (described above) was randomly assigned to colonies. Caged queens were introduced into colonies on 5 June and released on 7 June. Colonies were maintained as single brood-chamber units throughout the summer with boxes for honey storage being added above a queen excluder as needed. Fall management was according to normal commercial practice for the region, except that colonies did not receive any treatment for *Varroa*. All colonies were fed with fumagillin-medicated syrup and received Oxytetracycline treatments in powdered icing sugar according to label directions. Colonies were wintered in an indoor wintering building at the University of Manitoba campus and maintained under constant darkness at 5° C and standard ventilation conditions.

Colonies were sampled to assess *Varroa* mite levels using an alcohol wash to assess the number of mites per bee and moved inside the wintering building on 31 October, 2013. Winter survival of colonies was assessed when the colonies were brought out of the building on 17 April, 2014. Colonies containing the experimental queen and any number of workers were considered alive. The overwinter survival was calculated based upon the number of live colonies in spring (any number of bees with a live queen) divided by the number of live colonies in late fall. Data were analysed as described above for overall survival of asymptomatic colonies in the AFB challenge experiment.

Uniform starting populations of bees and *Varroa* mites were obtained by combining mite infested bees from several hives into large screened boxes (133 by 72 by 68 cm). Source colonies consisted of 30 *Varroa*-infested colonies from the University of Manitoba Apiary, 50 colonies that originated from Australian packages, and 30 colonies of *Varroa*-infested bees obtained from a local producer. On 5 June, frames of bees were shaken from the University of Manitoba colonies (25.7 kg) and Australian packages (70.1 kg) into two large cages. These were maintained in the wintering building in a cool dark room overnight to allow thorough mixing of the bees and mites from different source colonies. Six sample cups of workers were taken from different locations in each cage (a total of 4,941 bees). The following morning, these were weighed, counted and subjected to alcohol wash,

to obtain an average weight per bee and to determine the number of mites per gram of bees for each cage. A mixture of 450 g of mites and bees was then shaken into each brood chamber with entrances screened, an experimental queen was added (in a cage). The inoculated colonies with queens were then left in the overwintering building overnight to settle before moving them out to the experimental yards on 7 June, releasing the queens and opening the hive entrances. In order to obtain the desired target starting populations of bees and mites a second inoculation was carried out one week later. Bees purchased from a local producer that had not been treated with acaricide were shaken into a single large cage on 13 June (51.8 kg of bees), sampled 14 times (a total of 4880 bees) and processed as described previously. An additional allocation of bees and mites were added to each colony to bring the starting population to 300 mites and 1 Kg of bees on 14 June. These bees were added to existing colonies on 14 June by pouring them into an empty hive top feeder that was placed on top of each colony with a piece of newspaper placed between the brood chamber and feeder. This was done to unite the additional bees to the colony in such a way as to minimize loss of the project queens.

Honey Production Assessment

Packaged bees (1 kg) imported from New Zealand were hived at three locations: Lethbridge, Alberta (48 colonies, 1 apiary), Beaverlodge, Alberta (96 colonies, 2 apiaries), and Glenlea, Manitoba (96 colonies, 2 apiaries) in May 2013. Original queens were removed from all packages and experimental queens were then introduced into each queenless package. Only colonies with laying experimental queens were included in the experiment. Colonies headed by queens of each of the four experimental stocks (MAS, FAS, BEN, and IMP) were distributed throughout the apiary and blocked in groups of four with one colony of each stock type randomly positioned within each block. Colony entrances at each site were rotated in such a way that colonies from each stock type faced different directions. The queens were confined to single brood-chamber hives following common commercial management practice, and given pre-weighed honey supers (combs containing honey) as required. Half of the colonies within each stock were randomly assigned treatment with an acaricide (Amitraz®) but since mites were intentionally at very low levels in the source colonies and this mite treatment had no measurable effects on honey yield it was not included as a factor in the analysis. The total amount of honey produced was evaluated for a total of 191 experimental colonies across the five sites, and only colonies that had their experimental queen for the duration of the summer were included in the honey production analyses (n=169). Total honey production per colony was determined by subtracting the weight of full honey supers, and subtracting the empty weight of the supers. Differences among queen stocks were analysed by ANOVA (Proc Mixed, REML, SAS) using apiary site as a blocking factor. Data were log-transformed prior to analysis.

On-line Methods References

1. Kuzyk, M. A. *et al*. Multiple reaction monitoring-based, multiplexed, absolute quantitation of 45 proteins in human plasma. *Mol. Cell. Proteomics* **8**, 1860-1877 (2009).
2. Swanson, J. A. *et al*. Odorants that induce hygienic behavior in honeybees: identification of volatile compounds in chalkbrood-infected honeybee larvae. *J. Chem. Ecol.* **35**, 1108-1116 (2009).
3. Cobey, S. W. Comparison studies of instrumentally inseminated and naturally mated honey bee queens and factors affecting their performance. *Apidologie* **38**, 390-410 (2007).
4. McCutcheon, D. Indoor wintering of hives. *Bee World* **65**, 19-37 (1984).

5. Anderson, D. L. & Trueman, J. W. H. Varroa jacobsoni (Acari: Varroidae) is more than one species. *Exp. Appl. Acarol.* **24**, 165-168 (2000).
